# Simulation Study of Ink Droplet Spraying Based on Sand 3D Printing

**DOI:** 10.3390/mi16060621

**Published:** 2025-05-25

**Authors:** Hailong Song, Ran Yan, Lei Xia, Qing Zhao, Qing Qiu

**Affiliations:** College of Mechanical Engineering, Chongqing University of Technology, Chongqing 400054, China; songhl@stu.cqut.edu.cn (H.S.); xialei@stu.cqut.edu.cn (L.X.); zhaoqing0102@gmail.com (Q.Z.); qiuqing1999@gmail.com (Q.Q.)

**Keywords:** sand mold 3D printing, volume of fluid model, droplet formation, numerical simulation

## Abstract

To address the challenge of imprecise micro-droplet formation control in piezoelectric jetting devices used in sand mold 3D printing and apply on-demand inkjet printing technology to sand mold manufacturing, this study first explains the working principle of a piezoelectric shear-mode printhead. A mathematical model of the droplet ejection process is then established based on Computational Fluid Dynamics (CFD). Building upon this model, numerical simulations of droplet generation, breakup, and flight are conducted by using the Volume of Fluid (VOF) model within the Fluent module of the Workbench 2020 R2 platform. Finally, under consistent driving conditions, the effects of key parameters—viscosity, surface tension, and inlet velocity—on the ejection process are investigated through simulation. Based on the results, appropriate ranges and recommended values for ink properties are determined. This study provides significant engineering value for improving the stability and precision of droplet formation in industrial sand mold 3D printing.

## 1. Introduction

Sand mold 3D printing is a groundbreaking digital manufacturing technology for producing sand molds [1]. It leverages droplet ejection techniques to construct complex three-dimensional molds layer by layer with high precision and efficiency [2,3]. In this process, inkjet technology precisely controls the ejection of adhesive in terms of location and amount, allowing for the efficient and accurate construction of complex three-dimensional molds [4,5]. To meet the requirements for printing speed and precision, the design of the printheads has evolved to be more precise and efficient [6]. Although the printheads used in piezoelectric inkjet technology are costly and have a long lifespan, they consume a significant amount of ink, resulting in a higher demand for ink than for the printheads themselves. Therefore, an ink supply system is used instead of integrated ink cartridges to better meet market demands [7]. Since the flow of ink in the pipeline is restricted, selecting ink with suitable properties is crucial to improving printing efficiency and image quality [8,9]. Variations in ink droplet speed and the generation of satellite droplets can lead to unwanted splashing and local quality changes in the sand mold during droplet deposition. The combination of numerical simulation, experimental analysis, and theoretical calculations can provide a detailed understanding of droplet formation and ejection in the inkjet 3D printing process [10,11]. Therefore, studying the droplet dynamics in the inkjet 3D printing process is of significant importance.

There are many models available for studying multiphase systems, but the Volume of Fluid (VOF) model has proven to have good correlation with experimental studies [12,13]. While numerous studies aim to understand droplet dynamics in the 3D printing process, research exploring droplet dynamics in the process of sand mold manufacturing is limited. For instance, Anas Bin Aqeel and colleagues studied the effects of nozzle geometry and fluid characteristics on droplet formation through CFD simulation [14], using the VOF method to simulate the impact of different contraction angles (such as 45°) and changes in the internal surface contact angle on droplet formation time and speed. They discovered that nozzles with a 45° contraction angle produce the most stable droplets at the highest speeds and increasing the contact angle extends formation time and reduces speed. Their research also identified optimal printing conditions for fluids with varying viscosities and surface tensions. Similarly, Liou and others used CFD and microfluidic visualization techniques to study droplet characteristics from a bent-mode piezoelectric printhead through internal digital codes [15]. They conducted experiments with commercial ink fluids and found high consistency between experimental and numerical data. They evaluated the relationship between the waveform of the piezoelectric printhead used in the VOF model and the resonant pressure and validated the simulation results with experimental data [16]. Shi Min proposed a Volume of Fluid method based on the study of droplet formation [17], predicting droplet volume and speed and clarifying the impact of ink characteristics on droplet formation, enhancing the precision and efficiency of inkjet printing. P.H. Chen and H.Y. Peng applied the finite difference and VOF methods to numerically simulate and analyze the parameters of piezoelectric inkjet printheads [18], studying the impact of these parameters on the performance of droplet generators and providing experimental results and performance evaluations, which serve as vital references for understanding the working principles of droplet generators and enhancing their performance. The studies mentioned primarily addressed the formation and control of droplets in inkjet printing through experimental validation and parameter analysis. However, they did not sufficiently explore how ink viscosity and surface tension vary under different environmental conditions. This lack of detailed research restricts a comprehensive understanding of ink behavior under various practical application conditions, which could impact the optimal formulation of ink and adjustments in inkjet printhead design.

## 2. Inkjet Simulation Modeling

### 2.1. Principles of Inkjet Technology

**Figure 1:**
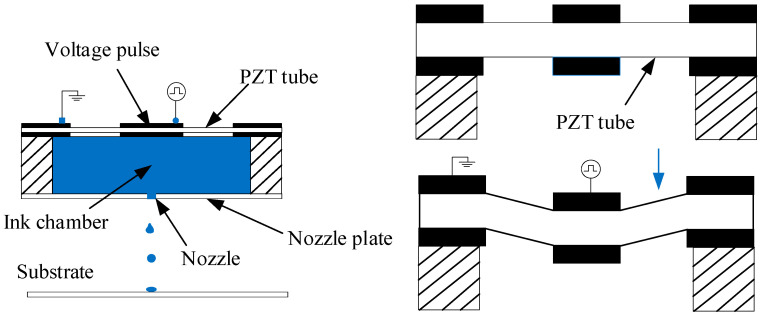
Inkjet printing principle diagram.

Piezoelectric inkjet technology is a technology that uses the properties of piezoelectric (PZT) ceramic elements to realize fluid injection. Shown in Figure 1 is the internal structure of the shear-type printhead in the volumetric piezoelectric printhead. There is no intermediate drive block or diaphragm; composed of piezoelectric ceramic sheet, nozzle, and ink chamber, the control circuit directly applies a voltage so that the piezoelectric ceramic sheet is subjected to downward shear action, thereby causing the ink to be a pressurized jet [19].

**Figure 2:**
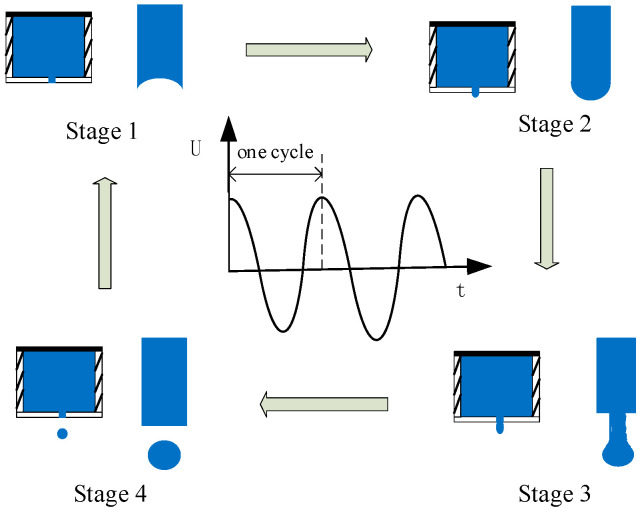
Ink droplet ejection process schematic diagram.

The single-cycle on-demand injection process of piezoelectric-driven droplet injection technology can be analyzed in four phases (Figure 2). The four phases correspond to exactly one cycle of control signal changes. The piezoelectric ceramic chip is not applied to the voltage drive signal (stage 1);the ink in the ink chamber needs a certain negative pressure so as not to slowly leak out from the nozzle holes onto the nozzle surface due to the capillary phenomenon and thus presents a “crescent-shaped surface” and remains in the ink chamber. When th ink needs to be sprayed, under control signal excitation, the piezoelectric ceramic sheet undergoes vibration deformation for extrusion from the ink cavity, the volume of the ink cavity contracts, and there are pressure changes (stage 2); this results in a pressure wave toward the nozzle, which drives the ink droplets to exit from the nozzle spray (stage 3 and stage 4).

### 2.2. Mathematical Modeling

The Fluent software(2020R2) application includes three types of multiphase flow models: the VOF model, the mixture model, and the Eulerian model. The VOF (Volume of Fluid) model accurately describes and tracks the interface between phases by calculating the volume fractions of each phase. This effectively simulates the interactions between different fluids, making it suitable for accurately capturing free surface flows and fluid interfaces [20]. For the ink droplet ejection process, it is important to note that the ink behaves as an incompressible laminar flow. Research primarily focuses on the gas–liquid interface and the droplet formation process. Given these considerations, the VOF (Volume of Fluid) model is effective in simulating the dynamic behavior and morphological changes of droplets during ejection. Therefore, employing the VOF model for this simulation is appropriate.

In the VOF model, a volume fraction function α is defined to describe the proportion of each fluid within a control volume. In a cell, let $\alpha_{q}$ be the volume fraction of a phase. When $\alpha_{q}=1$, it indicates that the cell is entirely occupied by one type of fluid; $\alpha_{q}=0$ indicates that it is occupied by another type of fluid. When $0<\alpha_{q}<1$, it signifies the coexistence of two fluids.

During operation, the ink solution used in the printhead is typically a dilute solution of polymers to maintain sufficiently low viscosity for jetting. Thus, these ink solutions can be modeled as Newtonian fluids. Furthermore, the density of the fluid does not change with pressure, making it incompressible. Based on this, the following assumptions can be established: (1) the ink is an incompressible Newtonian fluid; (2) the ink flows in a laminar state; (3) the properties of the ink remain constant during flow; (4) the solid boundary condition is a no-slip boundary. The control equations can be composed of the following set of equations [21].

In the VOF multiphase flow model, to accurately monitor the boundaries between fluid phases, the interface between different phases is tracked by solving one or more volume fraction equations. Therefore, for a phase, the equation for the volume fraction is shown in (1).

(1) $$\frac{\partial \alpha_{q}}{\partial t}+\nabla \cdot \left(\alpha_{q}u_{q}\right)=0$$

where $\alpha_{q}$ is the volume fraction of phase q, representing the ratio of the volume of phase q to the total volume of the control volume; $u_{q}$ denotes the velocity vector of the fluid in phase q; and ∇ is the divergence operator.

According to the equation of the conservation of momentum, the change in the momentum of each infinitesimal volume element in a fluid is equal to the sum of all forces acting on that element. The momentum conservation equation is shown in (2).

(2) $$\rho \left(\frac{\partial u}{\partial t}+u\cdot \nabla u\right)=-\nabla p+\mu \nabla^{2}u+F$$

where ρ is the density of the ink, u is the fluid velocity vector, p is the fluid pressure, μ is the dynamic viscosity of the fluid, ∇ is the gradient operator, $\nabla^{2}$ is the Laplace operator, and F represents volumetric forces such as gravity acting on the fluid. Together, these forces determine the acceleration and flow direction of the fluid elements.

The energy conservation equation is used to describe the changes in internal energy within a fluid, including heat conduction caused by temperature gradients, convection due to fluid motion, and heat generated by internal heat sources or viscous dissipation. The energy conservation equation is shown in (3).

(3) $$\rho c_{p}\left(\frac{\partial T}{\partial t}+u\cdot \nabla T\right)=k\nabla^{2}T+\dot{q}+\rho \dot{w}$$

where $c_{p}$ is the specific heat capacity of the fluid at constant pressure, T is the temperature, k is the thermal conductivity, $\dot{q}$ represents the heat source term per unit volume, and $\dot{w}$ is the heat generated per unit volume due to viscous dissipation.

The Finite Volume Method (FVM) is a numerical approach based on the laws of conservation, used for fluid flow calculations. It involves dividing the computational domain into multiple control volumes to ensure that the conservation laws are satisfied within each volume, thereby allowing for the precise calculation of fluxes at the boundaries. The SIMPLE algorithm, short for Semi-Implicit Method for Pressure-Linked Equations, is an iterative method designed to solve the Navier–Stokes and continuity equations [22]. It functions as a pressure–velocity coupling algorithm. The process begins with an assumed pressure field, followed by the calculation of the velocity field based on this assumption. Afterward, the pressure field is adjusted according to the newly calculated velocity field. This cycle continues until convergence is reached. In each iteration, the SIMPLE algorithm updates both the velocity and pressure fields, thus gradually approximating the accurate solution of the flow field.

### 2.3. Physical Modeling

**Figure 3:**
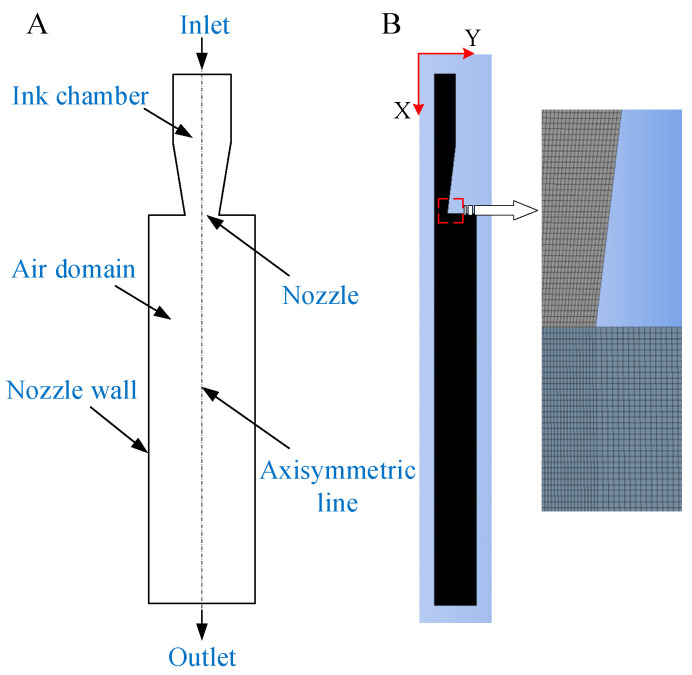
(**A**) Physical model diagram. (**B**) Mesh division.

To accurately simulate the droplet ejection process in a piezoelectrically driven micro-spray device, we employed a multi-physics coupled numerical model incorporating fluid dynamics, structural mechanics, and piezoelectric effects. The simulation used the VOF model within the Fluent module of Ansys 2020 R2 to capture the dynamic behavior of ink droplets, considering assumptions such as the incompressibility and laminar flow of the ink. To minimize the impact of the nozzle structure on the simulation results, a commonly used two-dimensional axisymmetric simplified model was selected to construct the physical model of ink droplet ejection, as shown in Figure 3A. Details on the dimensions of the ink droplet ejection physical model can be found in Table 1.

The physical model of droplet ejection primarily consists of two parts: the ink region and the air region. The ink region encompasses the internal structure of the piezoelectric printhead and is filled with ink, while the air region involves the path of the droplets as they are ejected from the nozzle and travel through the air until they reach the printing surface. The exit face of the nozzle represents the interface between the gas and the liquid, marking the initial point of contact between the ink and the air. The dynamic changes in this interface represent a critical focus in simulation analysis.

## 3. Numerical Simulation of Ink Droplet Ejection

The droplet ejection process can be viewed as a complex problem of free surface flow [23]. During this process, the liquid and gas come into contact, together forming a dynamically changing interface known as a free surface. This interface not only defines the boundary of the liquid flow domain but is also a critical factor to consider when solving flow field problems. The position and shape of the free surface are influenced by both fluid dynamics (such as flow velocity and pressure distribution) and fluid statics (such as surface tension).

When building models with simulation software, the fluid is considered the primary subject of study. In the model, the fluid and other physical fields such as structure are integrated into the same platform. By coupling the computational variables and related parameters of these physical fields, the comprehensive effects of multiple physical fields are realized. This method effectively solves the computational results under the interaction of various physical fields and accurately determines the position of the free surface. This paper uses the Fluent module on the Ansys 2020 R2 version Workbench platform for the simulation study of the droplet ejection process. It employs the built-in Design Modeler for model construction and the Workbench Meshing mixed mesh division tool for meshing.

### 3.1. Meshing Division

Mesh division plays a crucial role in the accuracy and computational efficiency of simulations. Generally, the finer and more numerous the mesh divisions, the higher the computational accuracy, but this also leads to greater consumption of computational resources [24]. Therefore, it is prudent to choose a quadrilateral, structured mesh that provides sufficient accuracy while relatively reducing computational complexity. With the nozzle centerline as the x-axis, the mesh model is illustrated in Figure 3B. The overall dimensions are small, with an area of 1.95 × 10^4^ μm^2^. The mesh consists of 71,835 cells and 72,862 nodes, and the mesh quality meets the accuracy requirements.

### 3.2. Boundary Condition Setup

**Figure 4:**
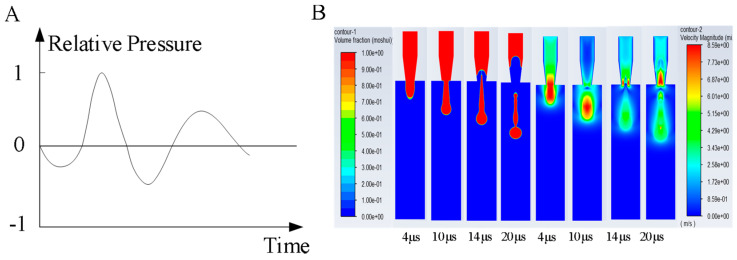
(**A**) Pressure wave at the ink chamber inlet. (**B**) Simulation diagram and velocity contour of the droplet formation process.

(1) Inlet conditions: In this simulation, the inlet conditions are emphasized. By using the pressure wave propagation theory proposed by Bogy et al. (as shown in Figure 4A), the voltage signal applied to the piezoelectric ceramics is transformed into ejection pressure.

(2) Outlet conditions: The outlet is set as a pressure outlet with zero backflow.

(3) Other conditions: The internal walls are set as fixed static walls (wall1), designated as “wall”. The outlet defaults to “interior”, and the other boundaries of the air domain default to “wall”, named “wall2”.

To accurately simulate the motion of gas–liquid interfaces, this paper employs a pressure-based VOF computational model and transient two-phase flow to simulate the droplet ejection process. Additionally, a laminar flow model is used, with no-slip boundary conditions set at the walls. The simulation also incorporates the Continuum Surface Force (CSF) model proposed by Brackbill et al. [25], while taking into account the effects of the contact angle at the walls.

## 4. Simulation Results and Data Analysis

### 4.1. Analysis of Droplet Morphology During the Ejection Process

By using the established simulation model, the simulation parameters were set as follows: viscosity μ = 1 cp, inlet velocity υ = 3 m/s, and surface tension of 72 dyn/cm. In Fluent software, a series of results were obtained through iterative calculations. These results display the phase changes and velocity distributions at different time points during the droplet formation process, as shown in Figure 4B, used to observe the morphology of a droplet at various moments.

The ink droplet ejection process can be divided into four main stages: droplet elongation, droplet necking, breakup and formation, and droplet ejection. As shown in Figure 4B, the initial stage of droplet formation occurs before 4 μs. At this time, the nozzle inlet is subjected to pulse pressure, and the piezoelectric ceramic deforms due to voltage drive, squeezing the ink chamber and forcing the ink to overcome its own surface tension, extruding and elongating to form a liquid column. At 10 μs, when the voltage is removed, the piezoelectric ceramic begins to revert to its original shape, the pressure inside the nozzle chamber gradually decreases, the liquid velocity slows, the amount of ink extruded reduces, the diameter of the ink column decreases, and necking occurs. Subsequently, as the piezoelectric ceramic resets, a negative pressure is generated inside the ink chamber. The ink column experiences opposing forces from above and below, along with the effects of gravity and its own surface tension, gradually coalescing into droplets that break and fall. At 14 μs, the droplet detaches from the nozzle plate and is ejected into the air domain. At this moment, the front end of the droplet reaches its maximum velocity and gradually detaches to form the main droplet. Due to uneven velocities, small droplets break off from the trailing ink column during the fall, forming satellite droplets.

The velocity contour of the droplet formation process is shown in Figure 4B. As the piezoelectric ceramic squeezes the nozzle chamber, the ink converges towards the nozzle orifice and its velocity rapidly increases until it reaches a maximum [26]. Afterward, as the movement speed of the piezoelectric ceramic decreases and it returns to its original position, the ink column begins to neck. Subsequently, the velocity begins to decrease until the column breaks.

### 4.2. Analysis of Ink Viscosity Effect

To determine the influence of viscosity on the droplet formation process according to the ink characteristics, the property parameters of typical ink adhesives at room temperature are utilized. The peak inlet velocity is set to 3 m/s with a frequency of 8 kHz. A single-factor simulation adjusts only the fluid viscosity, keeping all other parameters constant. Details for each group are shown in Table 2.

**Figure 5:**
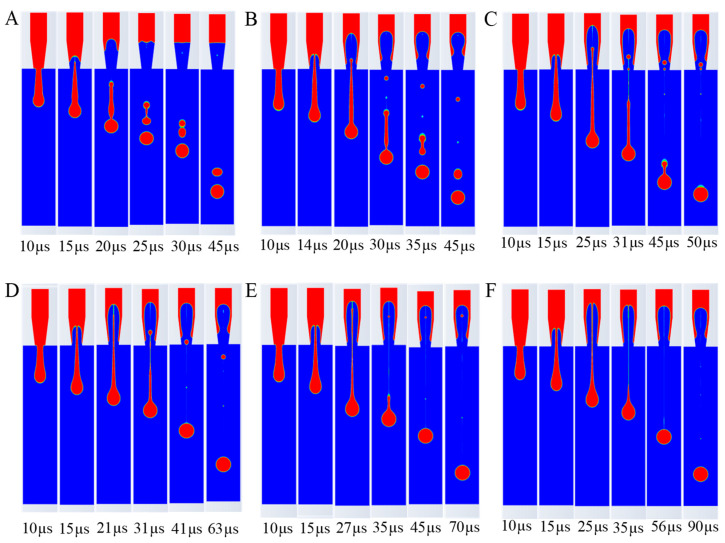
Influence of ink viscosity on ejection process. (**A**) 1 cp. (**B**) 5 cp. (**C**) 10 cp. (**D**) 15 cp. (**E**) 20 cp. (**F**) 30 cp.

The simulation results under various parameter conditions are shown in Figure 5 (the red region represents the liquid phase with a volume fraction of 1, while the blue region is air with a volume fraction of 0), leading to the following conclusions.

At a viscosity of 1 cp, the tail length post-breakup was the longest, with up to two satellite droplets forming. As viscosity increased, the number of satellite droplets decreased, indicating improved droplet coherence. At 15 cp, no satellite droplets were observed, and the droplet shape was optimal, suggesting that higher viscosity enhances droplet formation stability, though excessive viscosity (>15 cp) could impede ejection speed and increase clogging risk. This is because the high viscosity of the ink increases the viscous resistance between the ink molecules, requiring more energy to overcome this resistance, thus making droplet formation more difficult and slowing down the ejection speed. Moreover, too high a viscosity value can also lead to nozzle clogging. Therefore, excessively high values should be avoided when selecting ink viscosity.

**Figure 6:**
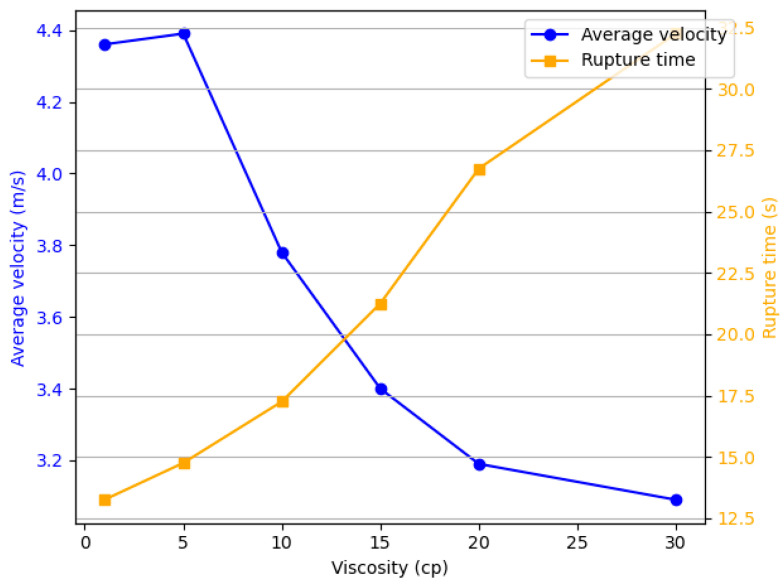
The relationship between the average velocity of the ejected fluid at different viscosities and the detachment time.

The droplet rupture time and average velocity indicators at different viscosities are shown in Figure 6. As the viscosity increases, the average droplet velocity exhibits a decreasing trend (from 4.36 m/s to 3.09 m/s, a decrease of 29%). When the viscosity exceeds 15 cp, the decrease in average velocity becomes less pronounced. The rupture time gradually lengthens withthe increase in viscosity (from 13.25 μs to 32.25 μs, an increase of 143.3%), which is relatively smaller compared with the increase in viscosity (290%).

### 4.3. Analysis of Ink Surface Tension Effects

As noted in the previous section, the number of satellite droplets increases as viscosity decreases. In addition to viscosity, surface tension also affects the droplet morphology. The following analysis examines the impact of surface tension variations on the droplet ejection state. The parameters for each group are listed in Table 3.

**Figure 7:**
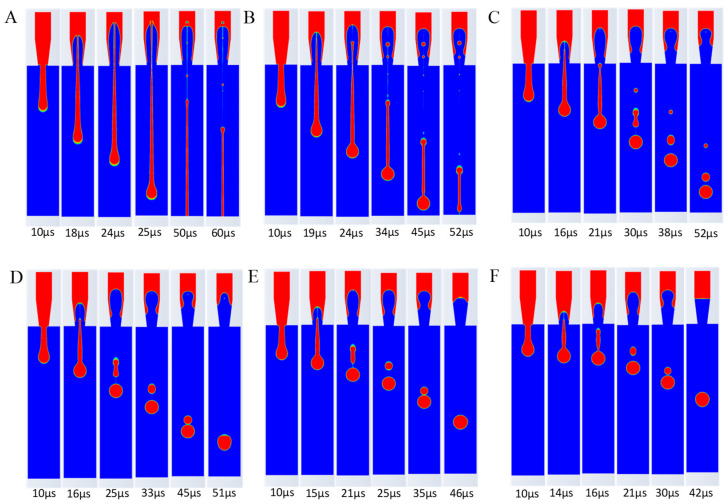
Influence of ink surface tension on ejection process. (**A**) 10 dyn/cm. (**B**) 30 dyn/cm. (**C**) 60 dyn/cm. (**D**) 72 dyn/cm. (**E**) 90 dyn/cm. (**F**) 120 dyn/cm.

From Figure 7, the following conclusions can be drawn: Firstly, as the surface tension increases, the trailing length of the ink droplet gradually shortens, and the jet necking effect becomes more pronounced, reducing the separation time between the main droplet and the trailing droplets. Secondly, the roundness and fullness of the droplets improve with the increase in surface tension. Lastly, as the surface tension increases, the number of satellite droplets decreases. Additionally, as shown in Figure 7D–F, the fusion time between the satellite droplets and the main droplet gradually shortens.

**Figure 8:**
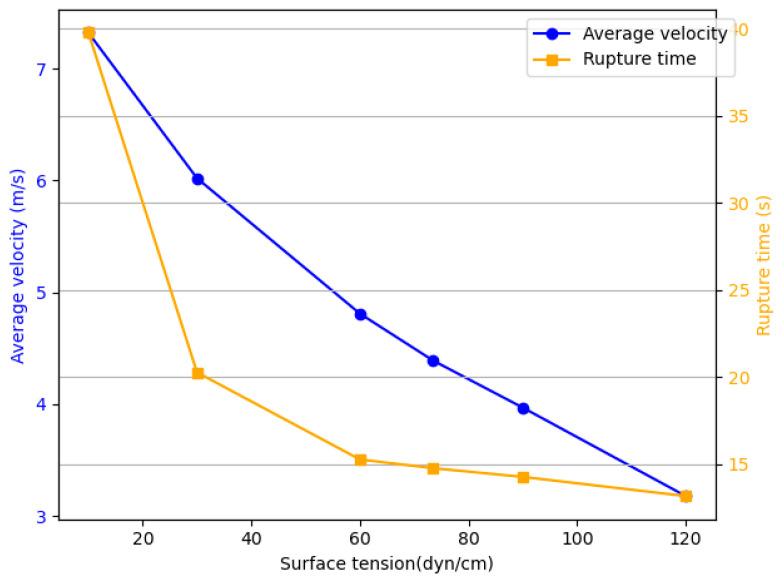
The relationship between the average velocity with different surface tensions and the detachment time.

As shown in Figure 8, the average ejection speed of the ink droplet shows a downward trend with an increase in surface tension. Specifically, the ejection speed of the droplet decreases from 7.32 m/s to 3.18 m/s, a reduction of 56%. The increase in surface tension significantly slows down the ejection speed of the droplet. At the same time, the rupture time also shortens significantly, decreasing from 39.75 μs to 13.15 μs, a reduction of 67%. This change is attributed to the contraction of the droplet surface as the droplet moves from the nozzle to form a free surface. During this contraction, the surface energy gradually decreases, and the surface tension does negative work on the droplet, causing it to release energy. The more negative work performed by the droplet, the more energy it releases, ultimately leading to a decrease in ejection speed.

### 4.4. Analysis of Ink Entrance Velocity Effects

Most of the nozzles on the market have ejection speeds between 4 m/s and 6 m/s, which corresponds to an ink inlet velocity of 2 m/s to 4 m/s. Therefore, different inlet velocity extremes are selected to analyze their impact on the ink droplet ejection state. The parameters for each group are listed in Table 4.

**Figure 9:**
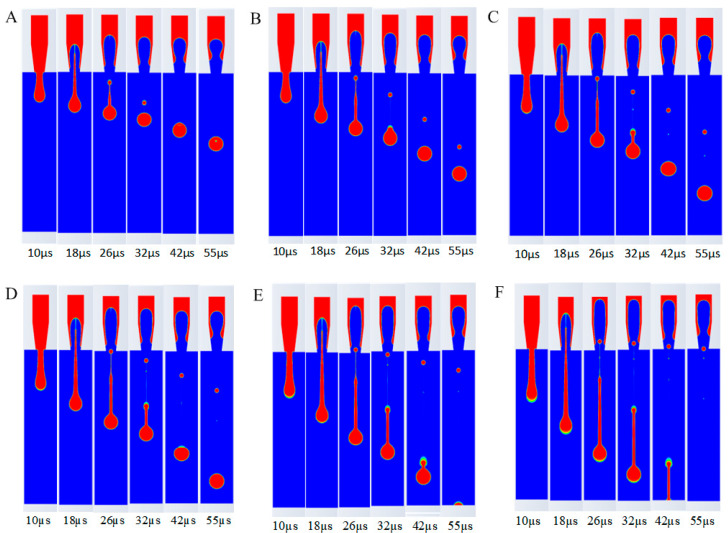
Influence of ink entrance velocity on ejection process. (**A**) 2.5 m/s. (**B**) 2.8 m/s. (**C**) 3.0 m/s. (**D**) 3.2 m/s. (**E**) 3.5 m/s. (**F**) 4.0 m/s.

From Figure 9, the following conclusions can be drawn: As the inlet velocity increases, the first tail formed by the main ink droplet gradually lengthens. Compared with the tail situation at 32 μs, it is evident that the main ink droplet forms a second tail, and as the velocity increases, the tail becomes longer, with the fusion time between the main ink droplet and the tailing droplets increasing. Finally, it can be observed that as the inlet velocity increases, the number of satellite droplets increases, and the flight speed of the droplets after ejection also increases, causing the satellite droplets to no longer merge with the main ink droplet. Therefore, the ink inlet velocity should not be set too high.

**Figure 10:**
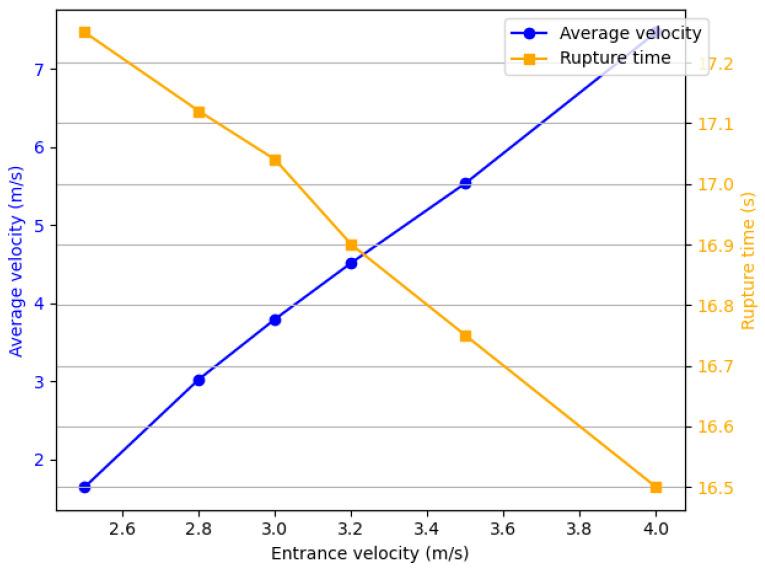
The relationship between the average velocity and the detachment time at different entrance velocities.

The relationship between the average speed and the rupture time at different inlet velocities is shown in Figure 10. As the inlet velocity increases, the average speed of the ink droplet shows an upward trend (rising from 1.65 m/s to 7.47 m/s, an increase of 352%). The detachment time gradually decreases with the increase in inlet velocity (from 17.25 μs to 16.5 μs, a decrease of 4.3%). It can be seen that the change in detachment time is relatively small compared with both the increase in inlet velocity (37.5%) and the increase in average speed (352%).

## 5. Conclusions

This paper applies piezoelectric inkjet technology to an ink supply system used in industrial 3D sand mold printing and proposes an active on-demand ink replenishment strategy. This approach aims to ensure timely and sufficient ink delivery during the printing process, thereby improving the stability and accuracy of droplet formation. The proposed method offers significant engineering value for enhancing the quality and reliability of sand mold fabrication in industrial applications. By using the VOF multiphase fluid dynamics analysis method and the Volume of Fluid (VOF) approach to track the deformation of the ink–air interface and the droplet formation process, a numerical simulation of the ink binder ejection process under a specific nozzle design is conducted. Additionally, to guide the selection of sand mold binder properties and nozzle design, this study investigates the effects of ink viscosity ranging from 1 cp to 30 cp, surface tension from 10 dyn/cm to 120 dyn/cm, and inlet velocity from 2.5 m/s to 4 m/s. The following conclusions are drawn:

(1) The higher the viscosity of the sand mold adhesive ink, the longer the droplet formation time, and the more likely it is to clog the nozzle. Therefore, it is advisable to use ink with reasonable viscosity, which should not be too high. The recommended value based on the simulation results is around 13 cp.

(2) The surface tension of the ink has a significant impact on the morphology of droplet formation. When the surface tension is low, it is more difficult for the droplet to form; when the surface tension is high, the droplet is more likely to form a shape close to a sphere, and the detachment time will also be reduced accordingly. This indicates that by adjusting the surface tension, the morphology and ejection characteristics of the droplets can be effectively controlled. The recommended value based on the simulation results is around 60 dyn/cm.

(3) The larger the ink inlet velocity, the longer the tail formed, and the more likely it is to generate a second tail. Additionally, satellite droplets do not merge with the main ink droplet, so excessively high inlet velocities should be avoided. The recommended value based on the simulation results is around 3.3 m/s.

## Figures and Tables

**Table 1:** Dimensions of the physical model of ink droplet injection.

Area	Diameter (μm)	Length (μm)
Ink chamber	30	50
Nozzle	18	50
Air domain	60	280

**Table 2:** Examples of viscosity variations in ejection simulation.

Index	Density (kg/m^3^)	Viscosity (cp)	Surface Tension (dyn/cm)
1	1200	1	72
2	1200	5	72
3	1200	10	72
4	1200	15	72
5	1200	20	72
6	1200	30	72

**Table 3:** Examples of surface tension variations in ejection simulation.

Index	Density (kg/m^3^)	Viscosity (cp)	Surface Tension (dyn/cm)
1	1200	10	10
2	1200	10	30
3	1200	10	60
4	1200	10	72
5	1200	10	90
6	1200	10	120

**Table 4:** Examples of entrance velocity variations in ejection simulation.

Index	Entrance Velocity (m/s)	Viscosity (cp)	Surface Tension (dyn/cm)
1	2.5	10	72
2	2.8	10	72
3	3.0	10	72
4	3.2	10	72
5	3.5	10	72
6	4.0	10	72

## Data Availability

Due to privacy or ethical restrictions, the data supporting the reported results cannot be made available.
